# Editorial: Global mental health among marginalized communities in pandemic emergencies

**DOI:** 10.3389/fpubh.2023.1249575

**Published:** 2023-07-21

**Authors:** Peter S. Navario, Nawaraj Upadhaya, Brian J. Hall, Lawrence H. Yang

**Affiliations:** ^1^Department of Public Health Policy and Management, New York University School of Global Public Health, New York, NY, United States; ^2^HealthRight International, New York, NY, United States; ^3^New York University School of Global Public Health, New York, NY, United States; ^4^NYU Shanghai Center for Global Health Equity, Shanghai, China; ^5^Department of Social and Behavioral Sciences, New York University School of Global Public Health, New York, NY, United States; ^6^Columbia University Mailman School of Public Health, New York, NY, United States

**Keywords:** coronavirus, COVID-19, mental health, marginalized/vulnerable population, pandemic

## Introduction

Chronic underinvestment in mental health at the global and national levels left us all vulnerable to the novel corona virus 2019 (COVID-19), which profoundly affects physical *and* mental health, with too few people, tools, and systems to marshal an effective response. In 2019, governments devoted an average of just over 2 percent of their health budgets to mental health, despite it being the *third* leading cause of the global disease burden (including self-harm and substance use), just after cardiovascular disease and cancer (1). Donors to low- and middle-income countries (LMIC) have performed arguably worse, allocating just US$220 million (0.3 percent) of US$67 billion in development assistance for health to mental health in 2021 (2). Exacerbating the deleterious effects of underinvestment, 93% of countries reported mental health service disruptions during the pandemic (3).

The mental health consequences of the pandemic include increased prevalence and severity of common mental disorders among people suffering from COVID-19 infection (4), among health care workers (5–7), and in the general population (8, 9). And while nearly everyone's mental health has been adversely impacted by the pandemic, there is growing evidence that marginalized communities have been disproportionately affected (9–11). This issue explores the impact that COVID-19 has had on the mental health of marginalized communities who typically bear a disproportionate burden of disease due to intersecting systems of discrimination and disadvantage.

## Aim/objectives of the Research Topic

This Research Topic adopts a novel perspective by examining how structural-level forces (i.e., the role of COVID-related policies, as well as pandemic-related disruption of essential services) impact the mental health of marginalized communities and provides examples of methods to bridge structural-level challenges (e.g., via telehealth). Specifically, this issue documents the prevalence of mental disorders within marginalized communities during the height of the COVID-19 pandemic, describes the adverse impact of pandemic prevention policies on mental health, evaluates program innovations to improve service access, provides digital and tele-health solutions to overcoming barriers to care and highlights the critical role played by civil society organizations and social safety nets in pandemic response.

The manuscripts and contributors featured are geographically diverse, with papers from Southeast Asia (2), South Asia (1), Africa (2), South America (1), the UK (1), and one scoping review with cross-regional coverage. The Research Topic also addresses a range of marginalized communities including displaced populations (2), migrant workers (1), the urban poor (2), adolescents and young adults in low-resource settings (1), ethnic minorities (2), and people who use drugs (1). We intend this Research Topic to make a distinct contribution to the literature and sharpen future mental health policy and research agendas to better address the mental health of marginalized communities during large-scale public health threats, focusing on but not limited to the pandemic.

## Themes highlighted in this Research Topic

### Elevated prevalence of mental health disorders among marginalized communities

Two papers herein document elevated prevalence of common mental disorders in marginalized communities during the pandemic. Ritsema and Armstrong-Hough report 30% prevalence of depressive symptoms among internally displaced Rohingya in Myanmar, with depression in the cohort highly correlated with post-displacement stressors including exposure to crime and violence. In the study by Koly et al. of urban slum dwellers in Dhaka, Bangladesh during the pandemic, 53% of participants had anxiety symptoms, and 43% reported mild to severe insomnia—significantly higher than previously reported in this population. Participant anxiety was associated with food insecurity, lack of access to clean water and sanitation, and fear of loved ones falling ill.

### Pandemic response: adverse consequences for mental health

This Research Topic explores the unintended adverse effects of COVID-19 containment policies such as isolation, social distancing, and the suspension of social services on the mental health of marginalized communities. In their ethnographic case studies of women living in the Sao Paolo exurbs, Bruhn et al. illustrate how pandemic policies like social isolation and social service discontinuation impacted women caregivers' mental health and overall wellbeing. Choe et al. scoping review shows the adverse psychological and physical effects of health elicited by psychosocial service disruption and isolation measures among people with opioid use disorder, including increased pain perception and reporting of mental health symptoms. Koly et al. found a significant protective association between the use of shared household facilities and lower rates of anxiety and insomnia among people living in urban slums, underscoring the adverse consequences of social isolation policies in Bangladesh. Finally, Van Bortel et al. conducted semi-structured interviews with ethnic minorities in the UK and found that poor communication from public health officials and the media increased anxiety about vaccine safety and confusion about pandemic restrictions.

### Digital and telehealth solutions' role in overcoming barriers to care

The Research Topic assesses several digital and telehealth interventions seeking to preserve or enhance access to mental health services for marginalized communities during pandemic emergencies. Munthali-Mulemba et al. evaluated the potential for telephone-delivered treatment to overcome structural barriers to mental health services (e.g., lack of providers and distance to health facilities) for adolescents and young adults in Zambia. Lee et al. assessed opportunities for digital and telephone-based mental health and psychosocial services to reach internally displaced persons (IDPs) in Myanmar during COVID-19. Finally, Liem et al. evaluated the delivery of the WHO Step-by-Step intervention utilizing a mobile phone application as part of a stepped care model for migrant workers in Macao, China.

### Social safety net as a critical component of pandemic preparedness and response

Several papers underscore the necessity of the social safety net in combination with mental health services for marginalized communities in the pandemic context. Van Bortel et al., reported on the diversity of pandemic experiences among ethnic minorities in the UK, including some who identified lacking culturally appropriate mental health services and economic support. Koly et al. demonstrated how the COVID-19 pandemic exposed significant gaps in the social safety net for the urban poor in Dhaka, Bangladesh, citing increased need for food aid, economic support, and improved administration of existing social net services. Finally, Bruhn et al. argue that mental health services in the absence of a strong social safety net are unlikely to be effective.

## Summary/recommendations

This Research Topic distinctly adds to a global literature by specifically elucidating impacts of COVID-19 on the mental health of marginalized communities. Sustained investment that is commensurate with worldwide disease burden at the global and national level in mental health along with relevant services is required to address today's unmet needs, and to prepare for future pandemics or other global crises (e.g., climate change). Effective, low-cost, and scalable mental health interventions exist and should be prioritized for rollout, particularly for the socially, economically, and geographically marginalized populations. The root causes driving incidence of mental disorders, such as access to quality education and healthcare, economic insecurity and respect for human rights, merit further investment as well. Secondly, mental health services require buttressing by a robust social safety net, and new low-cost, culturally appropriate, trauma-informed interventions must be developed for the marginalized communities. Two papers in this issue (Liem et al.; Lee et al.) demonstrate the critical role civil society plays in ensuring access to mental health and psychosocial support services and they must be engaged. Meaningful participation of marginalized communities and people with mental health conditions in governmental advisory group for pandemic response can help strengthen community based mental health systems. Amid the COVID-19 pandemic, the war in Ukraine led to another mental health crisis, which further highlights the necessity of strengthening mental health systems to be resilient and responsive to calamities as they arise (12). These emergencies provide an opportunity to build mental health systems that are inclusive, equitable and responsive to the needs and priorities of the marginalized communities. Whether it is pandemics, war or other crises, the time to address mental health capacity is now.

## Author contributions

PN drafted the editorial. LY, BH, and NU reviewed and edited the drafts. All authors contributed to the article and approved the submitted version.

## References

[1] GBD Compare. Retrieved from Institute of Health Metrics and Evaluation. (2023). Available online at: https://vizhub.healthdata.org/gbd-compare/ (accessed April 28, 2023).

[2] Financing Global Health. Retrieved from Institute of Health Metrics and Evaluation. (2023). Available online at: https://vizhub.healthdata.org/fgh/ (accessed April 28, 2023).

[3] World Health Organization. The impact of COVID-19 on Mental, Neurological and Substance Use Services: Results of a Rapid Assessment. Geneva: World Health Organization. (2020).

[4] Daugherty SE Guo Y Heath K Dasmariñas MC Jubilo KG Samranvedhya J. Risk of clinical sequelae after the acute phase of SARS-CoV-2 infection: retrospective cohort study. Bmj. (2021) 3:373. 10.1136/bmj.n1098 34011492 PMC8132065

[5] Vizeh M Qorbani M Muhidin S Javanmard Z Esmaeili M. The mental health of healthcare workers in the COVID-19 pandemic: a systematic review. J Diabetes Metab Disord. (2020) 19:1967–78. 10.1007/s40200-020-00643-9 33134211 PMC7586202

[6] Chutiyami M Cheong AM Salihu D Bello UM Ndwiga D Maharaj R. COVID-19 pandemic and overall mental health of healthcare professionals globally: a meta-review of systematic reviews. Front Psychiatry. (2022) 12:2600. 10.3389/fpsyt.2021.804525 35111089 PMC8801501

[7] Chigwedere OC Sadath A Kabir Z Arensman E. The impact of epidemics and pandemics on the mental health of healthcare workers: a systematic review. Int. J. Environ. Res. Public Health. (2021) 18:6695. 10.3390/ijerph18136695 34206264 PMC8296866

[8] Vindegaard N Benros ME. COVID-19 pandemic and mental health consequences: Systematic review of the current evidence. Brain Behav. Immun. (2020) 89:531–42. 10.1016/j.bbi.2020.05.048 32485289 PMC7260522

[9] Camara C Surkan PJ Van Der Waerden J Tortelli A Downes N Vuillermoz C. COVID-19-related mental health difficulties among marginalised populations: A literature review. Cambridge Prisms: Global Mental Health, (2023) 10:e2. 10.1017/gmh.2022.56 36843877 PMC9947635

[10] Hooper MW Napoles A Perez-Stable E. COVID-19 and racial/ethnic disparities. JAMA. (2020) 323:2466–67. 10.1001/jama.2020.8598 32391864 PMC9310097

[11] Brakefield WS Olusanya OA White B Shaban-Nejad A. Social determinants and indicators of COVID-19 among marginalized communities: a scientific review and call to action for pandemic response and recovery. Dis Med ublic Health Prep. (2022) 17:193. 10.1017/dmp.2022.104 35492024 PMC9237492

[12] Shi W Navario P Hall BJ. Prioritising mental health and psychosocial services in relief and recovery efforts in Ukraine. The Lancet Psychiatry. (2022) 9:e27. 10.1016/S2215-0366(22)00114-6 35390327

